# CCR2 limits inflammatory functions of CD8 TRM cells that impair recognition memory during recovery from WNV encephalitis

**DOI:** 10.1101/2024.09.17.613307

**Published:** 2024-09-21

**Authors:** Shenjian Ai, Artem Arutyunov, Joshua Liu, Jeremy D. Hill, Xiaoping Jiang, Robyn S. Klein

## Abstract

Central nervous system (CNS) resident memory CD8 T cells (T _RM_ ) that express IFN-γ contribute to neurodegenerative processes, including synapse loss, leading to memory impairments. Here, we show that CCR2 signalling in CD8 T _RM_ that persist within the hippocampus after recovery from CNS infection with West Nile virus (WNV) significantly prevents the development of memory impairments. Using CCR2-deficient mice, we determined that CCR2 expression is not essential for CNS T cell recruitment or virologic control during acute WNV infection. However, transcriptomic analyses of forebrain CCR2 ^+^ versus CCR2 ^-^ CD8 T _RM_ during WNV recovery reveal that CCR2 signalling significantly regulates hippocampal CD8 T _RM_ phenotype and function via extrinsic and intrinsic effects, decreasing the expression of CD103 and granzyme A and IFN-γ, respectively. Consistent with this, WNV-recovered *Cd8a* ^cre^ *Ccr2* ^fl/fl^ mice exhibit decreased recognition memory. Our findings highlight a neuroprotective role for CCR2 in limiting CD8 T cell-mediated neuroinflammation and cognitive deficits, providing insights into potential therapeutic targets for CNS infections.

## Full Text Availability

The license terms selected by the author(s) for this preprint version do not permit archiving in PMC. The full text is available from the preprint server.

